# Use Microfluidic Chips to Study the Phototaxis of Lung Cancer Cells

**DOI:** 10.3390/ijms20184515

**Published:** 2019-09-12

**Authors:** Fong-Yi Lin, Jin-Young Lin, Kai-Yin Lo, Yung-Shin Sun

**Affiliations:** 1 Department of Physics, Fu-Jen Catholic University, New Taipei City 24205, Taiwan; qaz54206@gmail.com (F.-Y.L.); aasd59472@gmail.com (J.-Y.L.); 2 Department of Agricultural Chemistry, National Taiwan University, Taipei 10617, Taiwan; kaiyin@ntu.edu.tw

**Keywords:** cell migration, phototaxis, microfluidic chips, reactive oxygen species, lung cancer cells

## Abstract

Cell migration is an important process involved in wound healing, tissue development, and so on. Many studies have been conducted to explore how certain chemicals and electric fields induce cell movements in specific directions, which are phenomena termed chemotaxis and electrotaxis, respectively. However, phototaxis, the directional migration of cells or organisms toward or away from light, is rarely investigated due to the difficulty of generating a precise and controllable light gradient. In this study, we designed and fabricated a microfluidic chip for simultaneously culturing cells and generating a blue light gradient for guiding cell migration. A concentration gradient was first established inside this chip, and by illuminating it with a blue light-emitting diode (LED), a blue light gradient was generated underneath. Cell migration in response to this light stimulus was observed. It was found that lung cancer cells migrated to the dark side of the gradient, and the intracellular reactive oxygen species (ROS) was proportional to the intensity of the blue light.

## 1. Introduction

Collective cell migration is involved in various physiological processes such as embryonic development [1,2], angiogenesis [3], and wound healing [4,5]. Particularly, this collective behavior plays important roles in the invasion and metastasis of cancer cells [4,6]. Many chemical and physical stimuli are known to induce directional cell migration, which is a phenomena termed “-taxis”. For example, chemotaxis, electrotaxis, and durotaxis describe how cells migrate in response to chemical, electric potential, and rigidity gradients, respectively. The chemotaxis of stromal cells and tumor cells is critical to cancer metastasis [7], and the abnormal chemotaxis of lymphocytes and leukocytes could lead to inflammatory diseases such as atherosclerosis and asthma [8,9]. Electrotaxis, or galvanotaxis, has been reported to be related to wound healing and the directional growth of cells and tissues during development and regeneration [10,11]. Durotaxis, or mechanotaxis, could affect stem cell differentiation [12], disease progression [13], and wound healing [14].

Phototaxis is the directional migration of cells or organisms (such as zooplankton and insects) toward or away from the stimulus of light. For example, phototrophic organisms can orient themselves to efficiently receive light for photosynthesis. Light–cell interactions have been reported to greatly affect the morphology, the production of reactive oxygen species (ROS), and the migration of various types of cells. Pattanaik et al. showed that the regulation of cellular morphology in *F. diplosiphon* was correlated with changes in light wavelength [15]. Under red light, *F. diplosiphon* cells are blue-green in color with a short and rounded shape. Conversely, under green light, *F. diplosiphon* cells are red in color with a longer and brick-liked shape. Ultraviolet (UV) and visible light were demonstrated to induce the production of ROS in cells, which could be a primary factor in skin damage [16,17]. Moreover, studies have found that the exposure of UVB radiation increased the generation of ROS and matrix metalloproteinases, which in turn triggered cell migration [18]. Although how cells respond to light of different wavelengths and intensities has been widely studied, the phototaxis phenomenon is rarely researched due to the difficulty in generating a precise and controllable light gradient. Recently, Lan et al. used a spatial light modulator (SLM) to project blue light with a quadratic intensity gradient on lung cancer cells [19]. It was observed that this light gradient could drive directional cell migration via the increment of the production of ROS [19]. Even though the SLM has been applied in generating light patterns for guiding cellular movement [20,21], this device is expensive, and other optical components (such as mirrors and lens) are required in the setup.

Microfabricated devices integrated with fluidic systems (such as pumps and valves) provide cells with diverse stimuli in a controllable manner so that their behaviors can be easily and systematically investigated. Compared with traditional cell culture dishes or microplates, only a very small amount of cells and reagents are consumed in such miniaturized devices [22]. Moreover, these in vitro fluid-circulating microchambers best mimic the in vivo microenvironment, where cells are subject to the fluidic shear stress of blood, urine, and all kinds of body fluids. Various microfluidic chips have been designed and fabricated for culturing cells and studying their chemotaxis and electrotaxis [23,24]. In the present work, a microfluidic chip was developed for simultaneously culturing cells and generating a blue light gradient for guiding directional cell migration. This light gradient was generated by shining light through a blue colorant-established concentration gradient, and the light intensity could be well controlled by adjusting the concentration of the blue dye. As observed, lung cancer cells migrated in the opposite direction to the light gradient. This phototaxis phenomenon was also shown to be related to the production of reactive oxygen species (ROS) in cells. With the aid of this microfluidic device, we hope to further investigate the mechanisms of light-induced cell migration.

## 2. Results and Discussion

### 2.1. Calculation and Simulation of Concentrations and Absorbance

**Figure 1:**
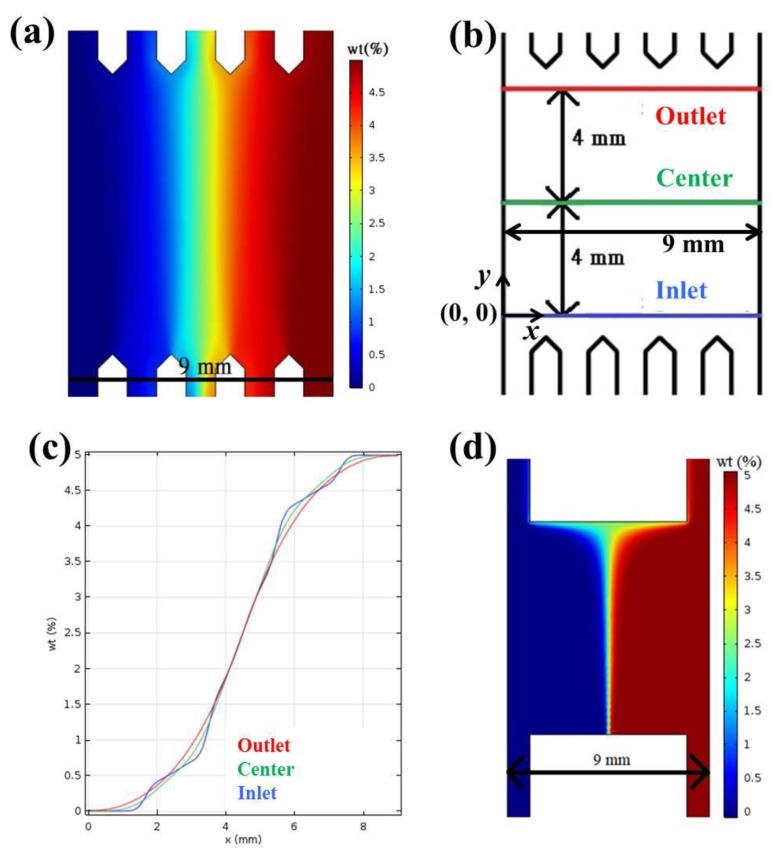
(**a**) Numerical simulation of Brilliant Blue FCF concentration inside the light-gradient chip. (**b**) The dimension of the light-gradient chip. (**c**) Concentration gradients along the three lines indicated in (**b**). (**d**) Numerical simulation of Brilliant Blue FCF concentration inside the H-shaped chip.

Figure 1a shows the COMSOL simulation of Brilliant Blue FCF concentration inside the light-gradient chip (the gradient area, see Section 3), where the concentrations in the two inlets are 0% and 5%, respectively. Concentration gradients could be observed in Figure 1c by drawing three lines across the gradient area, as indicated in Figure 1b. No matter whether the lines were near the outlet, in the center, or near the inlet, an almost linear region was achieved in x = approximately 2 to 8 mm, and a perfect concentration gradient with a slope of 1.33%/mm was generated in x = approximately 3.75 to 5.25 mm. Cells cultured underneath this perfect-gradient region were observed to ensure optimal light gradient. The H-shaped microfluidic chips were commonly used in chemotaxis experiments [25,26]. Figure 1d shows the COMSOL simulation of Brilliant Blue FCF concentration inside an H-shaped chip with the same parameters and settings. As demonstrated, the linear region in this chip was much narrower than that in the present chip, and this region also depended on its location, being the widest near the outlet and the narrowest near the inlet.

**Figure 2:**
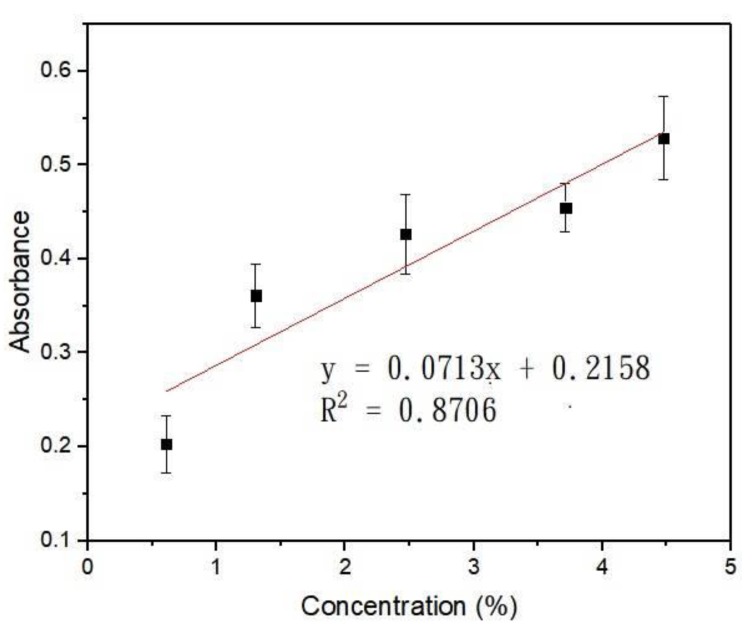
Relationship between Brilliant Blue FCF concentration and its absorbance to blue light.

At a digital intensity of 255, the powers of blue light underneath the gradient area were measured at x = 0.5, 2.5, 3.5, 4.5, 5.5, 6.5 mm, and y = 4 mm (the origin was indicated, see Figure 1b). The concentration at x = 0.5 mm was close to 0, so the power measured here was normalized to have a transmittance of 1. Table 1 lists the measured power, normalized transmittance, and absorbance (using A = −logT) at different positions. Figure 2 shows the absorbance plotted against the concentration derived from Figure 1c. The error bar indicated the SEM from three individual measurements. A linear correlation of R^2^ = 0.8706 suggested that within this concentration range, Beer’s law is valid. Light gradients of different slopes and magnitudes can be generated by flowing Brilliant Blue FCF of different concentrations into the light-gradient chip. Moreover, the same chip can be applied to create light gradients of different wavelengths [27].

### 2.2. Phototaxis of A549 Cells Induced by a Blue Light Gradient

**Figure 3:**
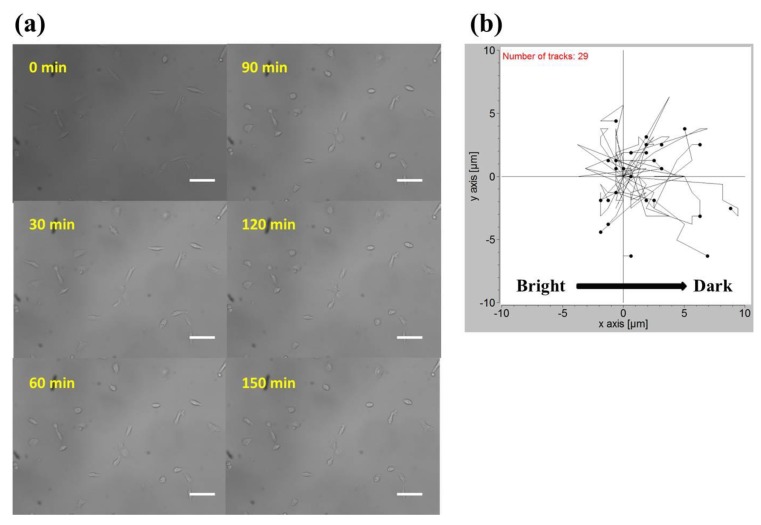
(**a**) Bright-field images of A549 cells exposed to the blue light gradient for 0, 30, 60, 90, 120, and 150 min. Scale bar = 100 μm. (**b**) Migration trajectories of 29 A549 cells after exposure to the blue light gradient for 150 min.

**Figure 4:**
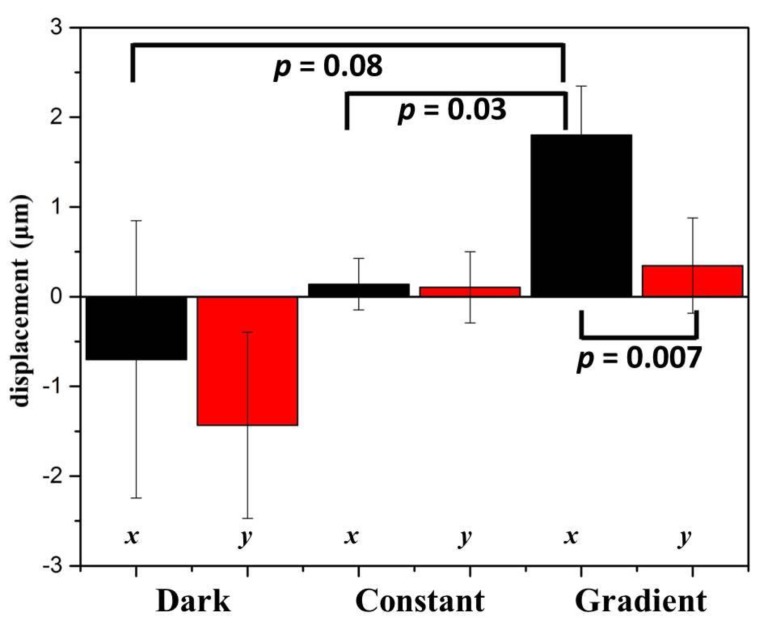
Mean displacements of A549 cells in directions parallel to the negative gradient (*x*) and perpendicular to the gradient (*y*) under different conditions. Student’s t-tests in the migration direction (*x*) between gradient and control groups and in the gradient group between the *x* and *y* directions were performed. *p* values were indicated.

The observation field of view (FOV) was about 0.7 mm × 0.7 mm in size and located in the center of the observation area. In Table 1, by plotting the power against the position, lung cancer A549 cells cultured in this region were subject to a blue light gradient of about 0.0239 (W/cm^2^)/mm. Figure 3a shows the bright-field images of this FOV after light stimuli of 0, 30, 60, 90, 120, and 150 min. The migration trajectories of 29 cells obtained from three independent experiments are shown in Figure 3b. Clearly, in the recording period of 150 min, A549 cells migrated away from the blue light, demonstrating negative phototaxis. Quantitatively, as indicated in Figure 4 (see the Gradient columns), the mean net displacements in the x (parallel to the direction of the negative gradient) and y (perpendicular to the direction of the gradient) directions were 1.800 μm and 0.347 μm, respectively. A549 cells appeared to migrate randomly in the y-direction because the error is larger than the mean value, and the migration speed in the x-direction was about 0.72 μm/h.

To further verify the effect of blue light gradient on cell migration, two control studies were conducted. In the first one, no light was illuminated on the chip, and the environment was completely dark. In the second one, 2.5% blue dye solutions were injected into the two inlets of the light-gradient chip. No concentration and light gradients were generated, and cells were exposed to a power of 0.129 W/cm^2^ or an intensity of 1160 J/cm^2^ for 150 min. In the case of no light (see the Dark columns in Figure 4), the error bars (1.540 μm and 1.038 μm in the x and y directions, respectively) were larger compared to the mean net displacements (−0.699 μm and −1.432 μm in the x and y directions, respectively). Therefore, without any light, A549 cells moved randomly in all directions with relatively longer migration distances. In the case of no gradient (see the Constant columns in Figure 4), A549 cells again migrated randomly without preferred directions, but small error bars (= 0.286 and 0.398 μm compared to mean net displacements of 0.140 and 0.105 μm in the x and y directions, respectively) suggested that these cells moved relatively shorter distances. From these results, it was concluded that blue light with a constant intensity inhibited the movement of cells, but a blue light gradient could induce directional cell migration. Similar results reported by Oh et al. showed that irradiation with blue LED inhibited the migration and invasion of mouse colon cancer CT-26 and human fibrosarcoma HT-1080 cells [28].

### 2.3. Dependence of the Intracellular ROS Level on the Intensity of Blue Light

**Figure 5:**
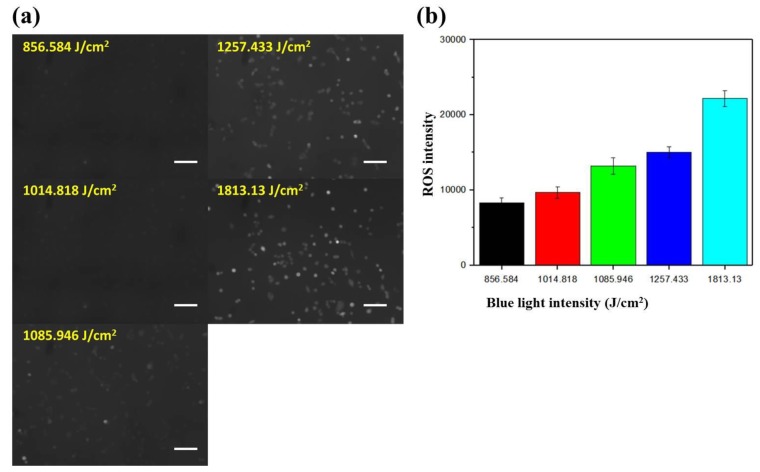
(**a**) Fluorescent images of A549 cells exposed to blue light intensities of about 857, 1015, 1086, 1257, and 1813 J/cm^2^. Scale bar = 100 μm. (**b**) Mean fluorescent intensities of cells in (**a**).

The intracellular ROS level increases as cells respond to environmental stresses such as light, heat, and electric field (EF). These chemical species (peroxides, superoxide, hydroxyl radical, singlet oxygen, and so on) regulate various cellular processes, including angiogenesis [29] and cell signaling [30]. To investigate the dependence of the intracellular ROS level on the intensity of blue light, cells cultured underneath the gradient area at x = 2.5, 3.5, 4.5, 5.5, 6.5 mm and y = 4 mm were observed. According to Table 1, the blue light intensities in these five regions were about 857, 1015, 1086, 1257, and 1813 J/cm^2^, respectively, for a total exposure period of 150 min. Figure 5a shows the fluorescent images of A549 cells under different blue light intensities. It was clearly seen that the cells became brighter and brighter as the light intensity was increased. The mean fluorescent intensity with SEM in an arbitrary unit is plotted against the light intensity in Figure 5b. The mean ROS intensities were 5264, 9665, 13197, 15049, and 22170 for light intensities of 857, 1015, 1086, 1257, and 1813 J/cm^2^, respectively. This result indicated that blue light stimulates the production of intracellular ROS in an intensity-dependent manner. Lockwood et al. found that dose-dependent ROS levels were generated in both normal and oral squamous carcinoma epithelial cells, but cumulative levels were higher and persisted longer in tumor cells [31]. It was also shown that blue light irradiation caused cell death in colorectal cancer by inducing ROS production and DNA damage [32]. Moreover, Wu et al. studied the correlation between cell migration and ROS under EF stimulation, and found that EF increases the production of intracellular ROS, which in turn accelerates directional cell migration. Therefore, we suggest that the blue light-generated ROS drives the directional migration of A549 cells, and these cells tend to migrate away from where more ROS were be produced.

### 2.4. Response of NIH/3T3 Cells to a Blue Light Gradient

**Figure 6:**
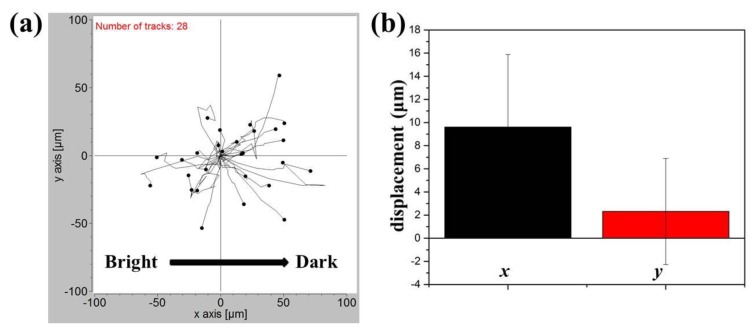
(**a**) Migration trajectories of 28 NIH/3T3 cells after exposure to the blue light gradient for 240 min. (**b**) Mean displacements of NIH/3T3 cells in directions parallel to the negative gradient (*x*) and perpendicular to the gradient (*y*).

For comparison, the response of NIH/3T3 fibroblasts to a blue light gradient was also investigated. Since almost all the NIH/3T3 cells died after the blue light exposure at a digital intensity of 255, the intensity was reduced to 65. In this case, cells cultured in the center of the observation area were subject to a blue light gradient of about 6.1 (mW/cm^2^)/mm. After an observation period of 4 h, the migration trajectories of 28 cells obtained from three independent experiments were shown in Figure 6a. Compared to A549 cells, NIH/3T3 cells appeared to move randomly with a longer migration distance (a few μm for A549 cells and a few tens of μm for NIH/3T3 cells). The mean net displacements with SEM in the x and y directions were indicated in Figure 6b. It seemed that NIH/3T3 cells also migrated away from the blue light with a mean net displacement of 9.614 μm in the x-direction. However, the error bar was relatively large, being 6.268 μm in the same direction. Therefore, the phototaxis of NIH/3T3 cells was not that obvious compared to that of A549 cells, and this phenomenon could be restrained by their active but random motility. The distinct phototactic behaviors of lung cancer cells could be due to their metastasis ability.

## 3. Materials and Methods 

### 3.1. Chip Design and Fabrication

**Figure 7:**
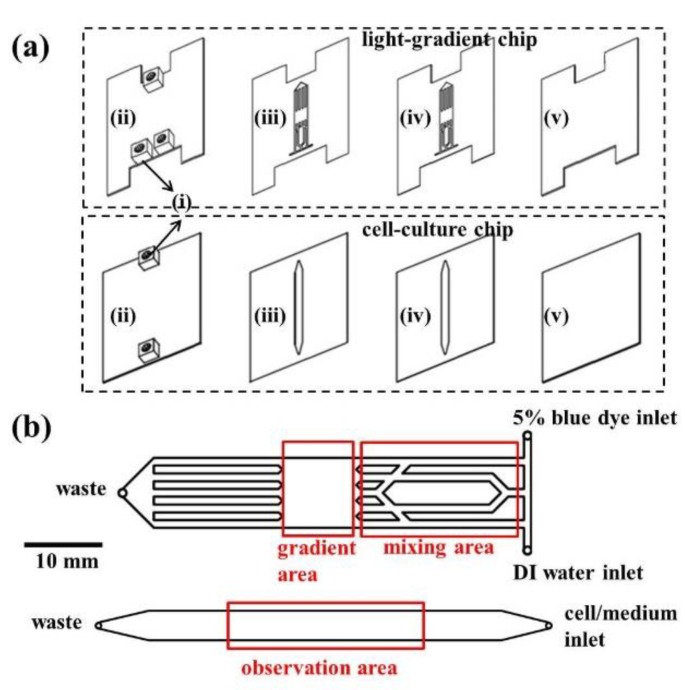
(**a**) Design of the microfluidic chip, including (i) adaptors, (ii) polymethylmethacrylate (PMMA) sheets with a thickness of 1 mm, (iii) double-sided tape with a thickness of 60 μm, (iv) double-sided tape with a thickness of 260 μm, and (v) PMMA sheets with a thickness of 1 mm. (**b**) Fluidic channels (thickness = 320 μm) of the light-gradient chip (top) and the cell-culture (bottom) chip.

The outline of the microfluidic chip is shown in Figure 7. The pattern was drawn in AutoCAD (ver. 2017, Autodesk, San Rafael, CA, USA) and then loaded into a CO_2_ laser scriber (ILS2, Laser Tools & Technics Co., Hsinchu City, Taiwan) to ablate desired patterns on polymethylmethacrylate (PMMA) substrates (HiShiRon Co., Tainan City, Taiwan) and double-sided tapes (8018, 3M, Paul, MN, USA). PMMA is widely used in the fabrication of microfluidic devices because of its transparency, low cost, and easy processing. This chip is composed of two parts: the top light-gradient chip and the bottom cell-culture chip. As shown in Figure 7a, the top and bottom four layers (two PMMA substrates with a thickness of 1 mm and two double-sided tapes with thicknesses of 60 and 260 μm) were bound together to form the light-gradient and the cell-culture chips, respectively. In each chip, the top PMMA layer had three or two small holes/adaptors serving as flow inlets and outlets. The gradient area in the light-gradient chip (Figure 7b top) is aligned with the center of the observation area in the cell-culture chip (Figure 7b bottom) by placing the two grooves in the top chip against the two adaptors in the bottom chip. The thickness of the cell-culture chamber is 320 μm, and cells were cultured on the PMMA substrate. The cytotoxicity of PMMA on cells was examined, and the result showed no observable cell viability change [33]. 

### 3.2. Calculation and Simulation of Concentrations and Absorbance

As shown in Figure 7b at the top, a Brilliant Blue FCF solution (Sigma, St. Louis, MO, USA, 5% *w*/*w* in DI water) and deionized (DI) water were injected from the two inlets. After passing the mixing area, a concentration gradient was generated in the gradient area due to continuous laminar flow mixing [34,35]. When a parallel blue light was illuminated on this gradient area, a blue light gradient could be generated underneath, considering the blue dye solution as the light blocker. The numerical simulation of chemical concentrations inside the light-gradient chip was performed using the commercial software COMSOL Multiphysics (ver. 5.3a, COMSOL, Burlington, MA, USA). The “Laminar Flow” and “Transport of Dilute Species” modules were used with the following parameters and settings: medium = water; diffusion coefficient = 2.8 × 10^−9^ m^2^/s for Brilliant Blue FCF [36]; inlet flow rate = 500 μL/h (set in each inlet); concentrations = 5% and 0% in the dye and water inlets, respectively. Beer’s law states that the absorbance (*A*) of a material to a specific light is proportional to the absorption coefficient of the material (*α*), the path length of the light (*x*), and the concentration of the material (*c*): *A* = *αxc*. Also, the absorbance is related to the transmittance (*T*) of the light to the material as *A* = −log*T*. By measuring the powers in different locations underneath the gradient chip and normalizing the transmittance to 1 for the region with the greatest power, we could relate the absorbance (*A*) to the concentration (*c*) to verify that the light gradient can be well controlled in both slope and magnitude.

### 3.3. Cell Preparation

Both the human lung carcinoma cells A549 and the fibroblasts NIH/3T3 were purchased from the Bioresource Collection and Research Center (BCRC) (Hsinchu City, Taiwan). Both types of cells were cultured in the complete medium consisting of Dulbecco’s modified Eagle medium (DMEM, Gibco, Waltham, MA, USA) and 10% calf serum (CS, Invitrogen, Carlsbad, CA, USA). Before seeding into the microfluidic chip, they were incubated in tissue culture polystyrene flasks (Corning, Corning, NY, USA) in 5% CO_2_ at 37 °C until 90% confluence.

### 3.4. Experimental System and Procedure

#### 3.4.1. Chip Assembly and Cell Injection

The microfluidic chip was assembled inside the laminar flow hood and UV-sterilized for 30 min. All inlets (two in the light-gradient chip and one in the cell-culture chip) were connected to syringes driven by syringe pumps (NE-300, New Era, Farmingdale, NY, USA). A total of 2.5 × 10^5^ cells suspended in 1 mL of DMEM with 10% CS was loaded into the cell-culture chip by injecting the solution from the inlet. The whole microfluidic chip was put on top of a transparent indium tin oxide (ITO) glass (Part No. 300739, Merck, Kenilworth, NJ, USA) which was connected to a proportional-integral-derivative (PID) controller (TTM-J4-R-AB, JETEC Electronics Co., Taichung City, Taiwan) for maintaining temperature at 37 ± 0.5 °C via feedback from a thermal couple (TPK-02A, TECPEL Co., New Taipei City, Taiwan)) clamped tightly between the heater and the chip. [37]. Cells attached to the surface in about 3 h, and then, fresh medium (DMEM with 10% CS) was continuously pumped into the chip at a flow rate of 30 μL/h for 18 h before blue light treatments. This continuous flowing of fresh medium provided cells with enough nutrition as well as removed waste from cells. 

#### 3.4.2. Blue Light Treatments

**Figure 8:**
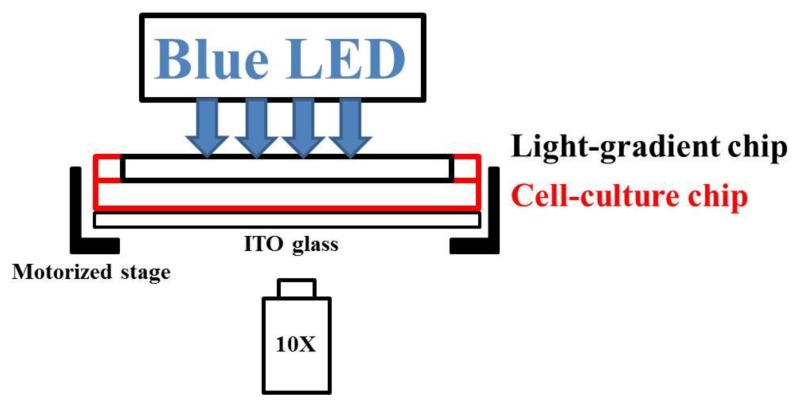
Side-view of the phototaxis system.

As shown in Figure 8, the microfluidic chip–ITO glass unit was mounted onto the motorized stage of an inverted microscope (FI40C, ESPA SYSTEMS Co., Hsinchu City, Taiwan). A planar blue light-emitting diode (LED) (Tytex Tech. Co., Kaohsiung City, Taiwan) with a center wavelength of 466 nm and a full width at half maximum of 40 nm was placed above the chip. Two syringes containing DI water and 5% blue dye solution were connected to the inlets of the light-gradient chip and driven by pumps at a flow rate of 500 μL/h. As soon as a stable concentration gradient was generated (about 1 h), the blue LED was illuminated on the light-gradient chip. Bright-field pictures of cells in the center of the observation area (Figure 7b bottom) were taken with a digital camera (60D, Canon, Tokyo, Japan) every 30 min for a total period of 2.5 h (A549 cells) or 4 h (NIH/3T3 cells). Digital intensities of 255 and 65 were used for A549 and NIH/3T3 cells, respectively, and the corresponding powers were measured with a power meter (DX-200, INS Enterprise Co., Taipei City, Taiwan).

#### 3.4.3. Cell Staining

In order to quantitatively measure the intracellular ROS level, the fluorescence-based ROS indicator H_2_DCFDA (Sigma, St. Louis, MO, USA) was used to stain the cells. This fluorophore has excitation and emission wavelengths of approximately 492 to 495 nm and 517 to 527 nm, respectively. After blue light treatments, a H2DCFDA solution of 10 μL in DMEM was continuously pumped into the cell-culture chip for 20 min at a flow rate of 20 μL/min. Afterwards, the chip was washed with DMEM for another 20 min at the same flow rate, and fluorescent images of cells (the same field of views as in the bright-field images) were captured with an inverted fluorescent microscope (FI40C, ESPA SYSTEMS Co., Hsinchu City, Taiwan) equipped with a digital camera (60D, Canon, Tokyo, Japan).

### 3.5. Data Analysis

Cell migration was tracked by taking bright-field images of cells located in the center of the observation area. This microscope was programmed to automatically repeat the observation in a time interval of 30 min. By using the ImageJ software (National Institute of Health, Bethesda, MD, USA), in each image, a polygon was drawn to enclose each cell, and the center of this cell could be located. Then, the migration trajectories were plotted using the Chemotaxis and Migration Tool software (National Institute of Health). For ROS quantification, the fluorescent intensities of the cells were analyzed again with the ImageJ software. A polygon was drawn to enclose each cell, and the mean intensity within the cell could be calculated. The background intensity was then subtracted to obtain the absolute signal. For each experimental condition, approximately 18 to 28 cells were selected from at least three independent experiments for data analysis. The standard error of the mean (SEM) was also calculated.

## 4. Conclusions

In this study, a microfluidic chip was designed and fabricated for studying the phototaxis of lung cancer A549 cells and NIH/3T3 fibroblasts. This chip was composed of two parts: a light-gradient chip and a cell-culture chip. As the blue LED illuminated the concentration gradient generated in the light-gradient chip, the cells cultured underneath were subject to a blue light gradient. A549 cells were found to migrate away from the blue light, showing negative phototaxis. The production of intracellular ROS was light intensity-dependent, suggesting that ROS could affect the directional cell migration under blue light stimulus. NIH/3T3 cells also exhibited phototaxis, but this phenomenon was somewhat restrained by their random motility. The present chip provides an in vitro platform for further investigating the mechanisms of light-induced cell migration.

## Figures and Tables

**Table 1:** Calculation of the absorbance of Brilliant Blue FCF to blue light (digital intensity = 255).

*x* position (nm)	0.5	2.5	3.5	4.5	5.5	6.5
Power (W/cm^2^)	0.320 ± 0.031	0.201 ± 0.0198	0.140 ± 0.0141	0.121 ± 0.0164	0.113 ± 0.0096	0.095 ± 0.0128
Transmittance	1 ± 0.097	0.630 ± 0.062	0.437 ± 0.044	0.377 ± 0.051	0.353 ± 0.030	0.298 ± 0.040
Absorbance		0.203 ± 0.053	0.361 ± 0.058	0.427 ± 0.073	0.455 ± 0.045	0.529 ± 0.076
Concentration		0.61%	1.30%	2.47%	3.71%	4.47%
